# Evaluation of the cardiovascular risk in patients with biliary stones: a descriptive cross-sectional study 

**Published:** 2018

**Authors:** Shermin Seddighi, Mohammad Esmaiel Ghidari, Amir Sadeghi, Mohammad Amin Shahrbaf, Mohammad Amin Mahmanzar, Saeede Saadati, Zahra Yari

**Affiliations:** 1 *Department of Cardiology, Taleghani Hospital, Shahid Beheshti University of Medical Sciences, Tehran, Iran.*; 2 *Gastroenterology and Liver Diseases Research Center, Research Institute for Gastroenterology and Liver Diseases, Shahid Beheshti University of Medical Sciences, Tehran, Iran.*; 3 *Faculty of Medicine, Shahid Beheshti University of Medical Sciences, Tehran, Iran. *; 4 *Basic and Molecular Epidemiology of Gastrointestinal Disorders Research Center, Research Institute for Gastroenterology and Liver Diseases, Shahid Beheshti University of Medical Sciences, Tehran, Iran. *

**Keywords:** Cardiovascular disease, Gallstones, Risk factors

## Abstract

**Aim:**

the aim of this study was evaluating the risk of cardiovascular disease in patients with gallstones.

**Background:**

Gallstones is the most common Biliary System disorder which its prevalence is increasing. On other hand, cardiovascular disease is the most common cause of mortality in the world. The causes and risk factors of cardiovascular diseases and gallstones are in common.

**Methods:**

In this descriptive cross-sectional study, patient with gallstones who hospitalized in Taleghani Hospital of Shahid Beheshti University of medical sciences or referred to its clinics in 2017, shared their demographic information and their underlying diseases with us. In addition, more data was collected with clinical examination, blood test, echocardiography and ultrasonography. Data was analyzed by SPSS vs21 software. In addition, online software was used for calculating Framingham and ASCVD risk score for cardiovascular diseases.

**Results:**

105 patients with gallstones and 105 healthy people participated in this study. There was no significant difference between these two groups for existence of main risk factors, but the average amount of ALT, AST, and ALP enzymes in patients with gallstones were significantly more than the control group (P value<0.05). The average amount of Framingham score was not significantly different between these two groups and the average score of ASCVD was statistically lower in our case group.

**Conclusion:**

The risk of cardiovascular disease in patients with gallstones is not significantly more than the general population.

## Introduction

 Gallstones is the most common biliary disorder that results in hospitalization (1). This disease imposes a significant economic-health burden on the health system even in western countries, where it is said that 10-20% of people in European and American countries have gallstones (2, 3). The cost of gallstones treatment in the United States is 6 billion dollars annually, which is the second most commonly reported post-reflux disease in terms of cost and burden of illness (4). It has also been estimated that about one million new patients with gallstones are diagnosed every year in the United States (5). The prevalence of gallstone is increasing, which can be due to reasons such as an increase in life expectancy and a change in dietary habits (6). Particularly after industrialization of societies and increase in risk factors for gallstones, the prevalence of cholesterol gallstones has increased significantly (2, 7). Many cases of gallstones do not show significant symptoms, on the other hand, about 25-50% of patients have complications and problems so that it is necessary to remove gallbladder in these people (5-8).

Cardiovascular disease (CVD) include coronary artery disease, cerebrovascular disease, peripheral arterial disease, and heart failure, which accounts for about one-third of world's mortality, about 18 million death per year, and estimate as the most common cause of mortality in the world (9-11). Studies have shown that by the year 2020, cardiovascular mortality in developed countries will continue to decline, but in developing countries, 85% of the world's population, CVD reaches its mortality peak (32 %) (11). Currently, there are about 15 million patients with heart disease in our country, and the average age of the disease has decreased to 42 years (12).

Recently, there have been few studies about association between gallstones and cardiovascular diseases. Although the causal relationship between gallstones and CVD is still unclear, previous studies indicated that the common characteristics in the pathophysiology of these two diseases causes association between these two diseases (13-15) ; one of these common characteristics is the accumulation of cholesterol in the gallbladder, as well as atherosclerosis in the arterial wall (16, 17). In addition to excessive cholesterol, other conditions, such as high blood pressure, diabetes, peripheral vascular disease, alcoholism, and chronic obstructive pulmonary disease are risk factors for both diseases (18-21).

Therefore, in view of the high prevalence of gallstones and its association with cardiovascular disease, by finding the relationship between these two diseases, patients with a history of gallstones may perform diagnostic and preventive measures for CVD earlier than the normal population. Therefore, the aim of this study is to evaluate the risk of CVD in patients with gallstones. 

## Methods

This is a descriptive-analytic cross-sectional study that evaluates the risk of CVD in patients with gallstones referred to surgical clinic of Taleghani Hospital in Tehran during 2017.

Before the study, patients were requested to complete and sign an informed consent to participate in this study. In this study, patients with gallstones, referred to surgical ward, and healthy individuals, matched in age and sex, were included. Based on the formula of the relevant sample volume and at a significant level of 0.05, about 85 people for each group was estimated. In order to increase the accuracy of the study, the minimum required sample size was 105 patients with gallstones referred to the surgical ward and 105 healthy individuals matched to patients in age and sex. Convenience sampling was used for the patient selection in both groups. 

The absolute risk of developing certain heart disease and death from it was also derived from the Framingham prospective study or the National Cholesterol Education Program (NCEP) (22-24). After entering the study, demographic information including age, sex, history of underlying diseases such as diabetes, hypertension, heart disease and history of smoking asked and recorded and Framingham values ​​was calculated for each person based on of seven major risk factors (age, sex, cholesterol, HDL-C, blood pressure, diabetes and smoking) (18, 22). Then, using the Framingham values ​​and tables provided by Framingham or the National Cholesterol Education Program (NCEP), we calculated the relative and absolute risk of CHD for each person. By existence of normal levels of the main risk factors (female gender, low age, low cholesterol levels, high HDL-C levels, lack of diabetes, normal blood pressure and non-smoker), It is possible to define a low-risk group and calculate relative risk; the probability of heart disease (cardiac events) in this group is relatively equal to 1 and in others, it was calculated as relative risk in compared to low risk group. 

Another method used to estimate the risk of CVD was the Atherosclerotic Cardiovascular Disease (ASCVD) (25). It also measures the risk of CVD and stroke by using variables such as age, sex, race, total cholesterol, HDL, chronic hypertension, diabetes, and smoking based on predicted percentages. Calculation of the ASCVD scale was also done using online software for each individual. We assessed Framingham and ASCVD scale by online software.

Quantitative anthropometric data such as blood pressure, weight and height were measured by resident of cardiology. Blood pressure was measured by a mercuric pressure gauge from the right arm for two times with 5 minutes’ interval, and the result was expressed as an average. Height and weight were measured on a standard scale. Anthropometric descriptive data such as physical activity were collected by a questionnaire from the patient by standard criteria (19), smoking, family history of heart disease, blood pressure and lipid disorders were measured too. Diabetes was defined as fasting blood glucose greater than 125 mg/dL or a specific diet or drug use. The presence of hypertension was defined as systolic pressure of more than 140 mmHg and diastolic blood pressure of more than 90 mmHg or antihypertensive therapy. Smoking was defined as + and - and the body mass index was determined by the BMI = kg/m^2^ equation.

Quantitative biochemical data was provided by testing the blood serum. Fasting blood was taken from an anticoagulant vein after 12 hours of fasting (9 P.M-9 AM) and blood was collected for analysis. Serum lipoproteins were determined for determination of lipid profile (total cholesterol, triglyceride and HDL-C). Cholesterol was calculated by the GPO-PAP and HDL-C enzymes measured by means of PTA-MgCl2 (biochemical) method. We used the Friedwold equation for measuring LDL-C.

Then echocardiography performed for each patient and EF and LV measurements and motion disturbances of the area walls calculated. Data was then compared with that of the control group, which was matched in age and sex.

In this study, all moral considerations were observed by the researchers. The entire procedure was approved by the Medical Ethics Committee of Shahid Beheshti University of Medical Sciences (Tehran, Iran: Protocol No. IR.SBMU.MSP.REC.1396.362). Participants were not obligated to complete the questionnaires, and diligence in relation to the information received from the participants was fully implemented and their information was used solely for the study. 

## Results

Finally, 105 patients with gallstones who referred to the surgical ward and 105 healthy individuals, matched in age and sex, participated in the study and successfully completed the required procedures. The demographic data of the participants in the two groups compared in table 1. BMI, weight and waist circumference in gallstones group were significantly higher than the control group (P value <0.05).

**Table 1:** The result of demographic data

Variable	Gallstones Group	Healthy Group	P value
Men	59 (56.19%)	47(44.76)	0.074
Women	46 (43.81%)	58 (55.24)	
Age	57.16± 18.44	58.43±18.53	0.616
Height (cm)	167.28±7.92	164.25±9.81	0.053
Weight (kg)	68.29±14.40	72.48±16.78	0.015
BMI* (Kg/m^2^)	24.39±4.79	26.76±5.36	0.001
Waist circumference (cm)	90.66±12.83	95.56±12.75	0.007

* Body mass index

**Table 2:** History of underlying disease

History	Number in control Group	Number in Gallstones Group	P value
Smoking	21	16	0.366
Cholecystectomy	0	23	<0.001
Dyslipidemia	28	46	0.031
TIA (Transient Ischemic Attack)	0	0	-
CVA (Cerebrovascular accident)	6	3	0.323
CHF (Congestion Heart Failure)	5	1	0.112
CAD (Coronary Artery Disease)	16	5	0.011
HTN (Hypertension)	31	33	1.00
DM (Diabetes Mellitus)	21	18	0.485

**Table 3:** Biochemical Data

Biochemical Variable	Control Group	Gallstones Group	P value
Total Cholesterol	32.41 ± 94.172	83/51 ± 17.172	0.906
Triglyceride	47 ± 98.141	64.60 ± 148	0.422
LDL	83.26 ± 87.85	89.32 ± 60.92	0.106
HDL	96.14 ± 47.47	44.7 ± 33.42	0.002
FBS	18.42 ± 18.111	82.59 ± 22.115	0.576
AST	12.79 ± 53.39	49.90 ± 07.72	0.006
ALT	55.89 ± 18.41	32.127 ± 89.87	0.003
ALP	76.380 ± 09.261	55.430 ± 89.464	<0.001

**Table 4:** Echocardiographic Abnormality

Echocardiographic Findings	Control Group	Gallstones Group	P value
Abnormal EF	3	0	0.075
Abnormal LV size	7	5	0.499
Abnormal RWMA	7	4	0.315

Participants in both groups were questioned about having some underlying diseases or some high-risk behaviors, which are major risk factors for cardiovascular problems. These risk factors include diabetes, chronic hypertension, history of cholecystectomy, smoking, dyslipidemia, history of TIA, CHF, history of CVA, and CAD. The number of people who had these risk factors in each group is presented separately in table 2. In fact, cholecystectomy and dyslipidemia was significantly higher in gallstone group (p value <0.05), furthermore, CAD was significantly higher in the control group. There is no significant difference between two groups in the presence of the main risk factors.

Quantitative biochemical data was obtained by serum test. The comparison of their mean in the two groups is presented in table 3. As shown in the table, the means of HDL, ALT, AST and ALP enzymes were significantly higher in patients with gallstones than in the control group (P value <0.05). Other findings did not differ between the two groups. The number of people with abnormal echocardiography is presented in table 4. There was no significant difference between the two groups in the echocardiographic findings.

For the purpose of calculating the risk of coronary artery disease for each person in the future, the Framingham and ASCVD score were used. As shown in Table 5, although the mean of Framingham score for those with biliary stones was higher than the control group, but difference between the two groups was not significant (P value = 0.972). However, comparison of mean ASCVD score for those with gallstones and the control group shows a significant difference between them (P value = 0.009). In fact, based on the ASCVD score, gallstones group are at a lower risk for atherosclerotic CVD.

**Table 5:** Risk Score of Cardiovascular Disease

Group		Score	P value
Framingham Score (mean±SD)			
	Gallstone	74/14±11/51	0.972
	Control	60/14±11.59	
ASCVD score (mean±SD)			
	Gallstone	13.62 ± 14.05	0.009
	Control	15.60 ± 18.67	

## Discussion

The aim of this study is to evaluate the risk of CVD in patients with biliary stones. Since many of risk factors for biliary stones such as high cholesterol and chronic blood pressure also considered as risk factors for CVD, it seems that patients with gallstones have a high risk for developing CVD. On the other hand, some studies have shown that the incidence of CVD in patients with biliary stones is higher than the general population regardless of age, gender and body mass index, which indicates that the higher risk of CVD in these patients may not be related to the risk factors of these two diseases alone. In general, previous studies have shown that those with any type of gallstones also have an increased risk for developing CVD, and even this increased risk does not relate to the severity of gallstones (26). 

For finding the relationship between biliary stones and CVD, we explore patients who had been referred to the Taleghani Hospital of Tehran due to the presence of biliary stones or were hospitalized in the department of gastroenterology and surgery. In order to compare these patients with the general population and generalize the results to the whole society, the equal number of healthy persons matched in age, sex and other factors were also included.

After analyzing the findings of this part of the questionnaire, it was found that patients with biliary stones had a higher mean BMI, weight and waist circumference than the control group (P value <0.05), but other individual characteristics did not differ between these two groups. Higher BMI and waist circumference in these patients can be attributed to different nutritional habits in group of patients and one of the factors that may predispose them to gallstones and heart disease. Interestingly, based on findings of the questionnaire, there was no significant difference between the two groups in terms of risk factors for CVD, so that some of the risk factors were higher in patients with gallstones and some others were more common in the control group. Our finding about BMI difference between two group was similar with recent study (15).

Also, in order to investigate the possible effects of biochemical agents in the blood in increasing the risk of CVD in these two groups, these factors were measured using a blood test. The findings showed a significant difference between the two groups in the mean of ALT, AST and ALP enzymes (P value <0.05) and the other findings did not differ between the two groups. Of course, higher levels of liver enzymes in patients with biliary stones are due to blockage from these stones and events and subsequent effects on the liver. In addition, evaluation of cardiac function by echocardiography and ECG did not show any significant difference between two groups.

Finally, we used Framingham and ASCVD scales to assess the risk of CVD. Using the major risk factors for CVD and online software, the risk of CVD was calculated separately for each of the participants, with the two mentioned scales. Contrary to the findings of previous studies, the average risk of CVD in patients with biliary stones was not significantly different from that of the general population (26-30). In fact, previous studies have shown that the risk of CVD in patients with bile ducts is more than the general population, which in our study did not result. In addition, comparing the two groups according to the ASCVD scale showed that even the risk of CVD was significantly higher in the control group than in patients with biliary stones (P value = 0.009). Although this finding is not consistent with the findings of previous studies, it can be due to changes in the lifestyle of patients with biliary stones for reducing their complications. In fact, improving lifestyle and reducing the risk of developing risk factors for people with biliary stones has accelerated the recovery and reduce the complications of their disease, which ultimately has a positive impact on the average risk of developing CVD in this group.

On the other hand, our sample size was lower than previous study; perhaps the differences in the results of the study are due to small size of population under study. Therefore, it is suggested that future studies should be done with a larger sample size.

Overall, the results of this study showed that the risk of CVD in patients with biliary stones is not significantly different from the general population. Of course, studies with a larger statistical society and a broader level of examinations are needed so that we can extend this result to the general population.

## Conflict of interests

The authors declare that they have no conflict of interest.
